# Supplementary material for: Kinetic features dictate sensorimotor alignment in the superior colliculus
Source: Nature. 2024 Jul 3;631(8020):378–85. doi: 10.1038/s41586-024-07619-2 (PMC11236723; doi:10.1038/s41586-024-07619-2)
Supplement: Supplementary file 1 — This file contains Supplementary Figs. 1 and 2 and Supplementary Tables 1 and 2 [file 41586_2024_7619_MOESM1_ESM.pdf]

nature portfolio

https://doi.org/10.1038/s41586-024-07619-2

## Supplementary information

# Kinetic features dictate sensorimotor alignment in the superior colliculus

In the format provided by the authors and unedited

| | N | RMP (Vm) | RI (MOhm) | Sag (mV) |
|---|---|---|---|---|
| *Direct* | 13 | -47.3 +- 0.9 | 543 +- 28 | 31.2 +- 5.9 |
| *Polysynaptic* | 13 | -50.2 +- 0.9 | 627 +- 24 | 136.6 +- 17.3 |
| *RAMP* | 10 | -52.6 +- 1.2 | 845 +- 90 | 39.7 +- 7.2 |
| *No activation* | 11 | -43.8 +- 0.9 | 599 +- 24 | 16.4 +- 1.2 |

| | AP threshold (mV) | AP amplitude (mV) | AP width (ms) | AHP Amplitude (mV) | AHP width (ms) |
|---|---|---|---|---|---|
| *Direct* | -37.7 +- 0.4 | 55.7 + 0.8 | 1.0 +- 0.0 | 13.3 +- 0.5 | 20.5 +- 1.8 |
| *Polysynaptic* | -32.4 +- 0.5 | 43.7 +- 1.1 | 1.4 +- 0.0 | 15.5 +- 0.4 | 52.5 +- 2.5 |
| *RAMP* | -37.0 +- 0.8 | 55.7 +- 0.8 | 1.3 +- 0.0 | 15.3 +- 0.8 | 26.9 +- 3.2 |
| *No activation* | -38.4 +- 0.6 | 61 +- 1.1 | 0.9 +- 0.0 | 15.8 +- 0.6 | 14.3 +- 1.4 |

| *+ 100 pA* | Num of AP | Frequency of burst (Hz) | Freq. Adaptation (%) | Freq. spikes 1 to 3 (Hz) | Delay to 1st AP (ms) | Time after last AP (ms) |
|---|---|---|---|---|---|---|
| *Direct* | 19.3 +- 1.2 | 38.3 +- 1.5 | 183.2 +- 7.2 | 51.6 +- 1.9 | 24.8 +- 1.4 | 206 +- 24 |
| *Polysynaptic* | 13.4 +- 1.2 | 18.2 +- 1.5 | 245.4 +- 45.7 | 26.2 +- 2.2 | 24.7 +- 2.8 | 332 +- 30 |
| *RAMP* | 18.4 +- 2.2 | 40.2 +- 2.8 | 172.1 +- 8.1 | 57.3 +- 5.1 | 43.2 +- 7.1 | 285 +- 35 |
| *No activation* | 33.7 +- 1.4 | 50.1 +-1.2 | 201.2 +- 6.2 | 74.1 +- 1.8 | 22.2 +- 2 | 123.2 +- 23 |

| *+ 200 pA* | Num of AP | Frequency of burst (Hz) | Freq. Adaptation (%) | Freq. spikes 1 to 3 (Hz) | Delay to 1st AP (ms) | Time after last AP (ms) |
|---|---|---|---|---|---|---|
| *Direct* | 3.8 +- 0.2 | 62.7 +- 4.2 | 103.1 +- 7.3 | 73.7 +- 4.8 | 3.9 +- 0.4 | 702 +- 16 |
| *Polysynaptic* | 6.8 +- 1.4 | 30.7 +- 3.9 | 72.2 +- 10.8 | 40.9 +- 5.2 | 6.6 +- 0.5 | 648 +- 25 |
| *RAMP* | 9.7 +- 2.1 | 42.1 +- 5.3 | 82.1 +- 9.1 | 49.4 +- 6.4 | 11.6 +- 1.3 | 672 +- 27 |
| *No activation* | 14.1 +- 1.8 | 102.4 +- 2.3 | 173.8 +- 15.7 | 135.4 +- 5.1 | 4.1 +- 0.5 | 647 +- 22 |

Supplementary Table 1. Electrophysiological properties of neurons recorded in acute brain slices and included in Figure 1.

| | N | RMP (Vm) | RI (MOhm) | Sag (mV) | Tuft width (um) | Tuft length (um) |
|---|---|---|---|---|---|---|
| *Superficial* | 24 | -50.1 +- 3.4 | 282 +- 44 | 28.2 +- 17 | 277 +- 160 (N =2) | 468 +- 3 (N =2) |
| *Upper Intermediate* | 12 | -41.9 +- 3.5 | 297 +- 97 | 3.8 +- 1.6 | 362 +- 49 (N =12) | 401 +- 44 (N =12) |
| *Lower Intermediate/ Deep* | 9 | -42.2 +- 3.4 | 164 +- 25 | 2.3 +- 1.0 | 379 +- 174 (N =3) | 452 +- 241 (N =3) |

| | AP threshold (mV) | AP amplitude (mV) | AP width (ms) | AHP Amplitude (mV) | AHP width (ms) |
|---|---|---|---|---|---|
| *Superficial* | -28.8 +- 2.0 | 21.5 + 2.9 | 0.7 +- 0.1 | 7.3 +- 1.2 | 31.0 +- 7.0 |
| *Upper Intermediate* | -26.0 +- 3.7 | 14.9 +- 2.4 | 0.7 +- 0.1 | 5.5 +- 0.9 | 26.5 +- 6.0 |
| *Lower Intermediate/ Deep* | -31.3+- 2.3 | 11.4 +- 0.8 | 0.6 +- 0.0 | 6.0 +- 0.7 | 41.0 +- 14.9 |

| *+ 100 pA* | Num of AP | Frequency of burst (Hz) | Freq. Adaptation (%) | Freq. spikes 1 to 3 (Hz) | Delay to 1st AP (ms) | Time after last AP (ms) |
|---|---|---|---|---|---|---|
| *Superficial* | 8.9 +- 2.4 | 11.4 +- 3.2 | 150.5 +- 63.0 | 23.7 +- 7.2 | 198.6 +- 59.6 | 49.9 +- 13.9 |
| *Upper Intermediate* | 13.4 +- 1.2 | 18.2 +- 1.5 | 245.4 +- 45.7 | 26.2 +- 2.2 | 24.7 +- 2.8 | 332 +- 30 |
| *Lower Intermediate/ Deep* | 18.4 +- 2.2 | 40.2 +- 2.8 | 172.1 +- 8.1 | 57.3 +- 5.1 | 43.2 +- 7.1 | 285 +- 35 |

| *+ 200 pA* | Num of AP | Frequency of burst (Hz) | Freq. Adaptation (%) | Freq. spikes 1 to 3 (Hz) | Delay to 1st AP (ms) | Time after last AP (ms) |
|---|---|---|---|---|---|---|
| *Superficial* | 19.8 +- 8 | 33.5 +- 12.1 | 238.0 +- 91.6 | 54.2 +- 17.9 | 3.9 +- 0.4 | 702 +- 16 |
| *Upper Intermediate* | 6.8 +- 1.4 | 30.7 +- 3.9 | 72.2 +- 10.8 | 40.9 +- 5.2 | 6.6 +- 0.5 | 648 +- 25 |
| *Lower Intermediate/ Deep* | 9.7 +- 2.1 | 42.1 +- 5.3 | 82.1 +- 9.1 | 49.4 +- 6.4 | 11.6 +- 1.3 | 672 +- 27 |

Supplementary Table 2. Electrophysiological and morphological properties of neurons recorded in whole-cell mode *in vivo* and included in Figure 2.

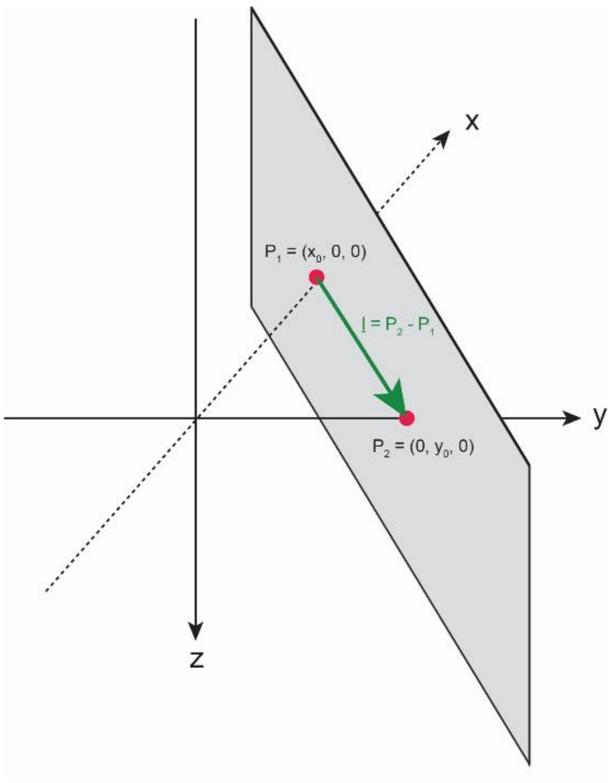


Supplementary Figure 1. Geometrical set-up of the visual stimulation screen in the laboratory's frame of reference.

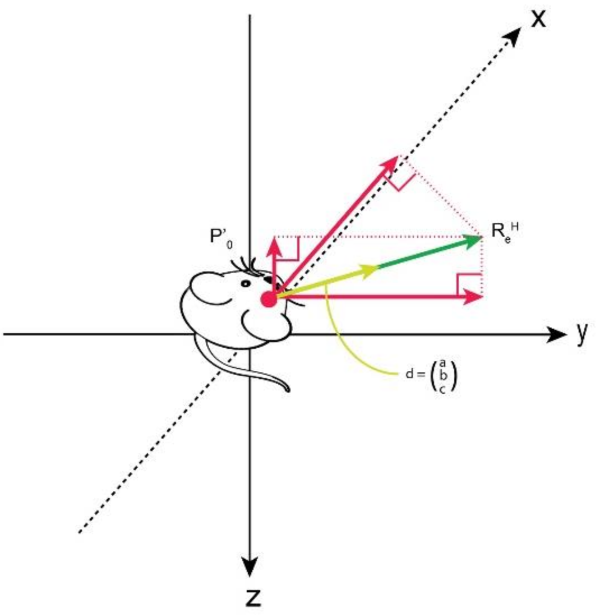


Supplementary Figure 2. Geometric set-up of the $r_e^H$ vector in the mouse's head reference frame, coinciding with the laboratory's reference frame at time zero.
