# Supplementary material for: Kinetic features dictate sensorimotor alignment in the superior colliculus
Source: Nature. 2024 Jul 3;631(8020):378–85. doi: 10.1038/s41586-024-07619-2 (PMC11236723; doi:10.1038/s41586-024-07619-2)
Supplement: Supplementary file 2 — Reporting Summary [file 41586_2024_7619_MOESM2_ESM.pdf]

nature portfolio

Corresponding author(s): Marco Tripodi & Ana González-Rueda

Last updated by author(s): May 3, 2024

Reporting Summary

Nature Portfolio wishes to improve the reproducibility of the work that we publish. This form provides structure for consistency and transparency in reporting. For further information on Nature Portfolio policies, see our Editorial Policies and the Editorial Policy Checklist.

Statistics

For all statistical analyses, confirm that the following items are present in the figure legend, table legend, main text, or Methods section.

n/a	Confirmed	
☐	☒	The exact sample size (n) for each experimental group/condition, given as a discrete number and unit of measurement
☐	☒	A statement on whether measurements were taken from distinct samples or whether the same sample was measured repeatedly
☐	☒	The statistical test(s) used AND whether they are one- or two-sided<br>*Only common tests should be described solely by name; describe more complex techniques in the Methods section.*
☐	☒	A description of all covariates tested
☐	☒	A description of any assumptions or corrections, such as tests of normality and adjustment for multiple comparisons
☐	☒	A full description of the statistical parameters including central tendency (e.g. means) or other basic estimates (e.g. regression coefficient) AND variation (e.g. standard deviation) or associated estimates of uncertainty (e.g. confidence intervals)
☐	☒	For null hypothesis testing, the test statistic (e.g. F, t, r) with confidence intervals, effect sizes, degrees of freedom and P value noted<br>*Give P values as exact values whenever suitable.*
☒	☐	For Bayesian analysis, information on the choice of priors and Markov chain Monte Carlo settings
☐	☒	For hierarchical and complex designs, identification of the appropriate level for tests and full reporting of outcomes
☐	☒	Estimates of effect sizes (e.g. Cohen's d, Pearson's r), indicating how they were calculated

Our web collection on statistics for biologists contains articles on many of the points above.

Software and code

Policy information about availability of computer code

Data collection

Data collection for whole-cell recordings was done using Clampex 10.6 (Molecular Devices).
Data collection for tetrode recordings was done using Axona DaqcUSB, Arduino Mega 2560 rev 3 boards.

All further details are described in the relevant paragraph of the method section of the manuscript.

Data analysis

Analysis routines were coded in Python v2.6 or in Igor pro v6.37 (Wavemetrics).
Cluster cutting for single unit determination was done with Tetrode INTerface (Tint) v4.2.6 (Axona).
The code for computational neuronal network model developed is available at https://github.com/AnaG-R/Visuomotor-model-SC.git

All further details are described in the relevant paragraph of the method section of the manuscript

For manuscripts utilizing custom algorithms or software that are central to the research but not yet described in published literature, software must be made available to editors and reviewers. We strongly encourage code deposition in a community repository (e.g. GitHub). See the Nature Portfolio guidelines for submitting code & software for further information.

Data

Policy information about availability of data

All manuscripts must include a data availability statement. This statement should provide the following information, where applicable:

- Accession codes, unique identifiers, or web links for publicly available datasets
- A description of any restrictions on data availability
- For clinical datasets or third party data, please ensure that the statement adheres to our policy

Pre-processed electrophysiological data has been uploaded to Zenodo (DOI: 10.5281/zenodo.11105001). Unprocessed data will be also made available upon request.

Human research participants

Policy information about studies involving human research participants and Sex and Gender in Research.

Reporting on sex and gender	Not applicable
Population characteristics	Not applicable
Recruitment	Not applicable
Ethics oversight	Not applicable

Note that full information on the approval of the study protocol must also be provided in the manuscript.

Field-specific reporting

Please select the one below that is the best fit for your research. If you are not sure, read the appropriate sections before making your selection.

☒ Life sciences ☐ Behavioural & social sciences ☐ Ecological, evolutionary & environmental sciences

For a reference copy of the document with all sections, see nature.com/documents/nr-reporting-summary-flat.pdf

Life sciences study design

All studies must disclose on these points even when the disclosure is negative.

Sample size	Sample sizes for each experiment were selected based on variance observed in similar previous studies and taking into account practical experimental considerations.
Data exclusions	All collected data from planned experiments were included in the analyses.
Replication	All data were successfully replicated in independent animals (>=5)
Randomization	Visual stimuli were presented in a semi-randomized manner.
Blinding	Analysis were blinded to data collection

Reporting for specific materials, systems and methods

We require information from authors about some types of materials, experimental systems and methods used in many studies. Here, indicate whether each material, system or method listed is relevant to your study. If you are not sure if a list item applies to your research, read the appropriate section before selecting a response.

Materials & experimental systems

n/a	Involved in the study
☐	☒ Antibodies
☒	☐ Eukaryotic cell lines
☒	☐ Palaeontology and archaeology
☐	☒ Animals and other organisms
☒	☐ Clinical data
☒	☐ Dual use research of concern

Methods

n/a	Involved in the study
☒	☐ ChIP-seq
☒	☐ Flow cytometry
☒	☐ MRI-based neuroimaging

Antibodies

Antibodies used	Primary antibodies:<br>Chicken anti-GFP (Aves Labs, GFP-1020, 1:2000)<br>Rabbit anti-RFP (Rockland, 600-401-379, 1:2000)<br>Secondary antibodies:<br>Alexa Fluor 488 donkey anti-chicken (Jackson ImmunoResearch, 703-545-155, 1:1000)<br>Cy3 donkey anti-rabbit (Jackson ImmunoResearch, 711-165-152, 1:1000)<br>Alexa Fluor 488 conjugated streptavidin (Invitrogen, 1:2000)
Validation	Both antibodies have been validated by the manufacturers and have been widely used by the scientific community in previous published studies.<br>In this study, we used the antibodies to assess viral expression. To evaluate specificity, we compared fluorescence between injection site and other brain areas and between injected and uninjected animals.

Animals and other research organisms

Policy information about studies involving animals; ARRIVE guidelines recommended for reporting animal research, and Sex and Gender in Research

Laboratory animals	The following mouse strains were used: WT C57BL/6 (The Jackson Laboratory: strain #000644), Pitx2-CRE::Tau-LSL-Flp0-INLA (derived from Pitx2-CRE and Tau-LoxP-STOP-LoxP-Flp0-INLA mice, provided by Prof. James Martin and Prof. Silvia Arber respectively), Pitx2-CRE::Rosa-LSL-tdTomato (derived from Pitx2-CRE and Rosa-LoxP-STOP-LoxP-tdTomato mice, #007914, The Jackson Laboratory) and or Vgat-CRE (#016961, The Jackson Laboratory).<br>Both sexes of mice were used for anatomical experiments while only males were used for behaviour. Experiments in adult animals were performed on mice aged between 8 and 12 weeks. All experimental procedures were performed in the MRC-LMB Animal Facility. Animals were group-housed in a 12 hours light/dark cycle (7 a.m. to 7 p.m.), with temperature controlled at 19-23 C, humidity controlled at 45-65%, and with food and water ad libitum except during food restriction periods.
Wild animals	No wild animals were used in this study.
Reporting on sex	All details are described in the relevant paragraph of the method section of the manuscript.
Field-collected samples	No collected field samples were used in this study.
Ethics oversight	All animal procedures were conducted in accordance with the UK Animals (Scientific procedures) Act 1986 and European Community Council Directive on Animal Care under project license PPL PCDD85C8A and approved by The Animal Welfare and Ethical Review Body (AWERB) committee of the MRC-LMB

Note that full information on the approval of the study protocol must also be provided in the manuscript.
